# Comparison of Presumed Geographic Retinal Dysplasia Between a Cavalier King Charles Spaniel and a Labrador Retriever

**DOI:** 10.1002/ccr3.72373

**Published:** 2026-04-03

**Authors:** Shaile Gehrke, Christine D. Harman, Amanda L. Jacobson, Gail McRae, András M. Komáromy

**Affiliations:** ^1^ Department of Small Animal Clinical Sciences, College of Veterinary Medicine Michigan State University East Lansing Michigan USA; ^2^ Leader Dogs for the Blind Rochester Hills Michigan USA

**Keywords:** autofluorescence, confocal scanning laser ophthalmoscopy (cSLO), geographic retinal dysplasia, microvascular anomalies, OCT angiography (OCTA), optical coherence tomography (OCT)

## Abstract

Geographic retinal dysplasia is inherited in the Cavalier King Charles Spaniel. This detailed multimodal imaging description of an affected dog demonstrates that the presumed dysplastic retina area consists of a thickened retinal nerve fiber layer, similar to plaque‐like lesions previously reported in other breeds, such as the Labrador Retriever.

## 1 Introduction

Retinal dysplasia (RD) is defined as the abnormal differentiation of the retina [1, 2, 3, 4, 5]. True RD may result from failure of the inner and outer layers of the optic cup to be in contact during embryogenesis, resulting in retinal detachment [6, 7, 8]. In dogs, the most common RD form is characterized by multifocal folds and rosettes in retinas that are otherwise mostly attached to the underlying retinal pigment epithelium [9, 10]. Larger RD lesions are defined as geographic and may develop in regions of partial retinal detachment [4, 11, 12]. Dysplastic changes typically develop in the canine retina at 45–50 days of gestation and generally can be diagnosed before 10 weeks of age, when the retinal layers are fully developed [1, 2, 3, 4, 5, 6, 7, 8, 9, 10, 11].

Retinal dysplasia can be inherited or acquired. For example, Labrador Retrievers and Samoyeds may be affected by oculoskeletal dysplasia (OSD) or dwarfism associated with RD (drd) [13, 14]. This condition is caused by mutations in either *COL9A3* (drd1 in the Labrador Retriever) or *COL9A2* (drd2 in the Samoyed) [13, 14].

Geographic RD‐like lesions that develop later than 10 weeks of age were described in some canine breeds, such as Golden Retrievers, German Shepherds, and Labrador Retrievers [11, 13]. By routine ophthalmoscopy, these cannot be differentiated from true geographic RD, and they are being diagnosed as such [15, 16]. However, high‐resolution imaging in affected Labrador Retrievers, Golden Retrievers, and German Shepherds revealed that at least some of these geographic RD‐like lesions consist of inner retinal plaques with tubular rosettes and vesicle‐like structures [11, 13, 17, 18]. The pathogenesis of these retinal plaques has not been defined; they may be acquired, but a genetic predisposition has not been ruled out.

Geographic RD is recognized as an inherited condition in the Cavalier King Charles Spaniel (CKCS) [15, 16]. The breeding recommendations by the Genetics Committee of the American College of Veterinary Ophthalmologists (ACVO) for RD‐affected CKCS are similar to most other canine breeds: While dogs with geographic RD should not be bred, dogs with focal/multifocal RD or retinal folds can still be bred (“breeder option”) [15]. The Hereditary Eye Diseases (HED) Committee of the European College of Veterinary Ophthalmologists (ECVO) states that breeding is optional for dogs affected by focal/multifocal or geographic RD unless the breed club issues different advice [16]. According to the *Blue Book on Ocular Disorders Presumed to Be Inherited in Purebred Dogs*, published by the ACVO Genetics Committee, of the 17,092 CKCS examined between 2019 and 2023, 1.6% had geographic RD, while 3.2% had retinal folds [15]. Even though these prevalences are higher than in the Labrador Retriever with 0.4% geographic RD and 0.9% retinal folds [15], no detailed morphological and clinical reports on geographic RD in the CKCS have been published to the best of our knowledge. It is unknown if these lesions represent true RD or are similar to the inner retinal plaques that develop later than 10 weeks of age in Labrador Retrievers, Golden Retrievers, and German Shepherds [11, 13, 17, 18]. To fill this knowledge gap, our retrospective study aims to describe high‐resolution imaging of geographic RD in a CKCS using confocal scanning laser ophthalmoscopy (cSLO), optical coherence tomography (OCT), and OCT angiography (OCTA). We compared our findings with the images of a Labrador Retriever with unilateral inner retinal plaques, since this condition has been previously characterized [11, 13, 17, 18].

## 2 Case Report

### 2.1 Clinical History

*Case 1‐Cavalier King Charles Spaniel (CKCS):* A 15‐month‐old intact female CKCS (body weight = 7.1 kg) was referred for advanced imaging following the identification of a geographic RD‐like lesion during a Companion Animal Eye Registry (CAER) examination. The dog exhibited no visual deficits and was otherwise healthy, with no evidence of systemic disease or medications.

*Case 2‐Labrador Retriever:* A 15‐month‐old intact male Labrador Retriever (body weight = 27.3 kg) purpose‐bred for a guide dog program presented for advanced imaging. The primary veterinarian identified a retinal lesion during a routine ophthalmic examination, although the dog exhibited no visual deficits. Physical examination revealed no evidence of systemic disease. Routine DNA testing showed that the dog was not a carrier of the *COL9A3* mutation responsible for osd1/drd1 [14].

Neither dog received a detailed ophthalmic examination before 4 months of age.

### 2.2 Ophthalmic Examination Findings

A comprehensive ophthalmic examination of the CKCS revealed Schirmer Tear Test values of 15 mm/min in the right eye (OD) and 16 mm/min in the left eye (OS) (Color Bar Schirmer Tear Test; Katena Products Inc.), with intraocular pressures of 16 mmHg OD and 15 mmHg OS (TonoVet Plus; Icare Finland Oy). Fluorescein staining of the cornea was negative in both eyes (I‐Glo Fluorescein Sodium Ophthalmic Strips; Jorvet). Tropicamide 1% ophthalmic solution (Akorn Inc.) was administered to achieve pharmacologic mydriasis. Slit‐lamp biomicroscopy (Kowa SL‐17 Portable Slit Lamp; Kowa Company Ltd.) revealed mild distichiasis on the inferior palpebra OD and a crescent‐shaped vitreal opacity dorsonasally in the vitreous OD. Indirect ophthalmoscopy (Keeler All Pupil II headset; Keeler Instruments; Pan Retinal 2.2D condensing lens; Volk Optical) identified a horseshoe‐shaped hyporeflective lesion OD and a focal, circular depigmented lesion in the ventronasal non‐tapetal fundus OS.

Genetic eye screening of the Labrador Retriever followed pharmacologic mydriasis with tropicamide 1% ophthalmic solution (Akorn Inc.). Slit‐lamp biomicroscopy did not reveal any abnormalities of the anterior segment OU. Fluorescein staining of the cornea was negative in both eyes. Indirect ophthalmoscopy revealed a focal, circular hyporeflective lesion in the dorsonasal tapetal fundus OS.

### 2.3 Ocular Fundus Imaging

#### 2.3.1 Preparation and Anesthesia Protocol

Both dogs underwent general anesthesia for OCT, OCTA, and cSLO imaging. Pre‐anesthetic bloodwork, including venous blood gas (VBG) and packed cell volume/total solids (PCV/TS), was normal. Premedications included ondansetron (1 mg/kg PO; Aurobindo Pharma USA Inc.), dexmedetomidine HCl (2–3 μg/kg IV; Zoetis Inc.), and butorphanol tartrate (0.2 mg/kg IV; Dechra Veterinary Products LLC). Induction of general anesthesia was performed with propofol (3–10 mg/kg IV; PropoFlo 28; Zoetis Inc.) and midazolam (0.2 mg/kg IV; Avet Pharmaceuticals Inc.). The dogs were intubated and maintained on isoflurane inhalant anesthesia (Isothesia; Henry Schein).

#### 2.3.2 Imaging Protocol and Techniques

Following pharmacologic mydriasis with tropicamide 1% ophthalmic solution (Akorn Inc.) and ocular surface anesthesia with proparacaine HCl 0.5% ophthalmic solution (Alcon Laboratories Inc.), the ocular fundus was imaged using the RetCam Envision with 130° wide‐angle lens (Natus Medical Inc.). Non‐ophthalmic ultrasound gel (Aquasonic 100; Parker Laboratories Inc.) was applied before the lens was placed on the cornea. These images were taken of the CKCS before and of the Labrador Retriever during general anesthesia, the latter following high‐resolution imaging.

Pharmacologic mydriasis was achieved with tropicamide 1% (Akorn Inc.) and phenylephrine HCl 10% (Lifestar Pharma LLC) ophthalmic solutions for high‐resolution fundus imaging. Spectral domain OCT, OCTA, and cSLO imaging (Spectralis HRA + OCT2 with OCTA module; Heidelberg Engineering Inc.) were performed under general anesthesia. Two conjunctival stay sutures were placed near the dorsal and ventronasal limbus (4–0 polypropylene; Covetrus North America) to control the eye position during imaging. A Barraquer eyelid speculum maintained the palpebral fissure open. Balanced salt solution (BSS; Alcon Laboratories Inc.) was applied every 30 s to prevent corneal drying. As previously reported, OCT and OCTA scans were acquired with a diode laser (820 nm wavelength), using either a 30° (OCT and OCTA) or 55° lens (OCT) [19]. Confocal SLO images were acquired with either a 30° or 55° wide‐field lens. The diode laser was used to capture regular infrared fundus images, while a solid‐state laser emitting at 488 nm wavelength was used to capture autofluorescent (blue reflectance) fundus images [19]. Analysis of the images was performed with the Heidelberg Eye Explorer software (HEYEX, Heidelberg Engineering Inc.).

### 2.4 Results

This section only describes abnormal or clinically relevant findings in detail; unmentioned structures were within normal limits.

#### 2.4.1 Retinal Camera Findings

**FIGURE 1:**
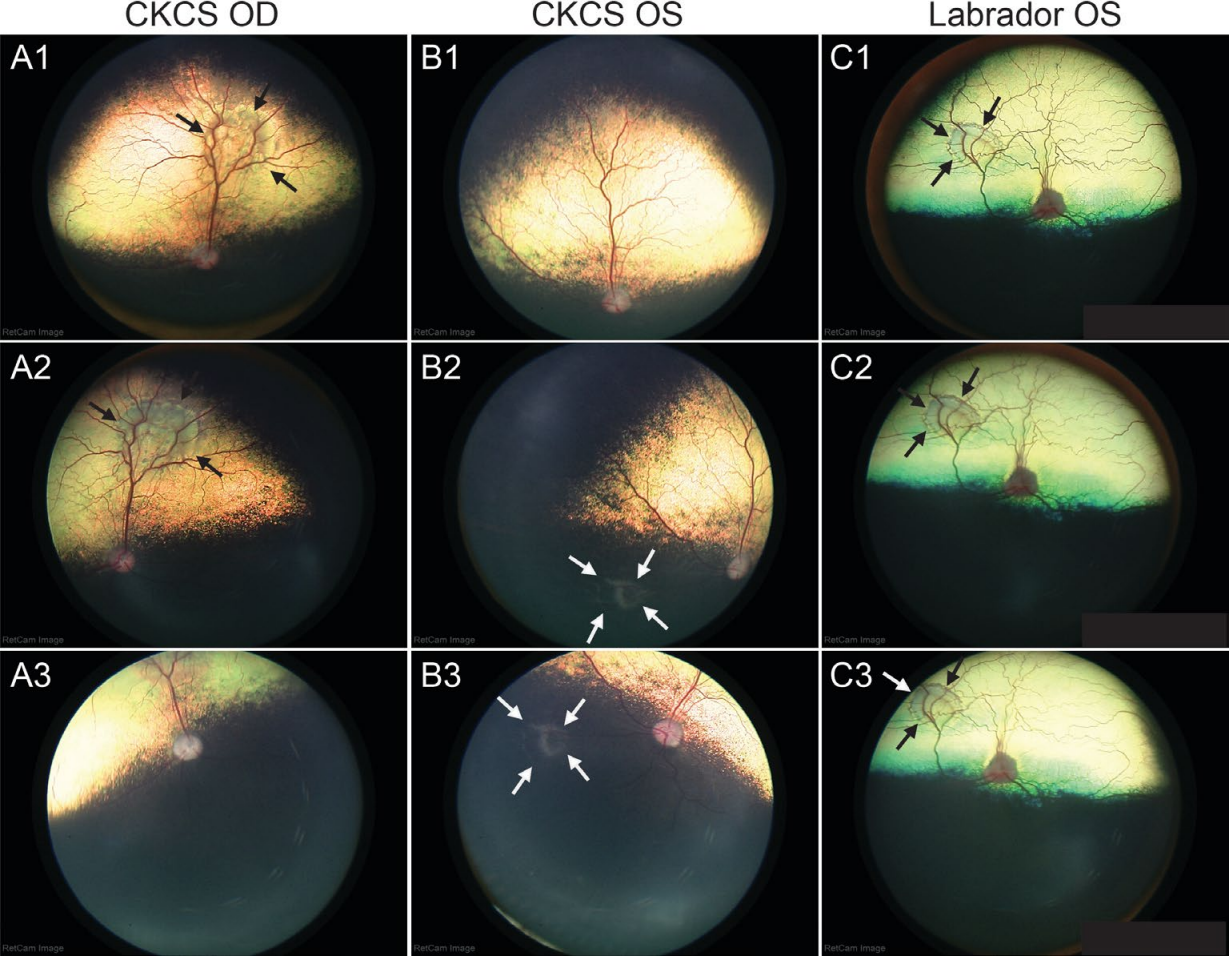
Fundus photographs of geographic RD‐like lesions in the CKCS and the Labrador Retriever. (A) The right eye (OD) of the CKCS shows a horseshoe‐shaped, hyporeflective lesion along the dorsal venule in the tapetal region (black arrows in A1 and A2). The non‐tapetal fundus OD appears normal (A3). (B) The left eye (OS) of the CKCS demonstrates a normal tapetal fundus with no significant abnormalities (B1). A focal, circular hypopigmented region is observed ventronasally in the non‐tapetal fundus OS, suggestive of geographic RD‐like lesion (white arrows in B2 and B3). (C) The OS of the Labrador Retriever contained a focal, circular, hyporeflective region dorsonasally in the tapetal fundus, consistent with a geographic RD‐like lesion (black and white arrows in C1, C2, and C3). CKCS, Cavalier King Charles Spaniel, OD, right eye; OS, left eye; RD, retinal dysplasia.

Retinal camera imaging of the CKCS revealed a horseshoe‐shaped hyporeflective lesion along the dorsal venule OD (Figure 1A). A focal, circular, depigmented lesion was observed ventronasally in the non‐tapetal fundus OS (Figure 1B). Retinal camera imaging of the Labrador Retriever revealed a focal, circular hyporeflective lesion in the dorsonasal tapetal fundus OS (Figure 1C).

#### 2.4.2 cSLO Autofluorescence Findings

**FIGURE 2:**
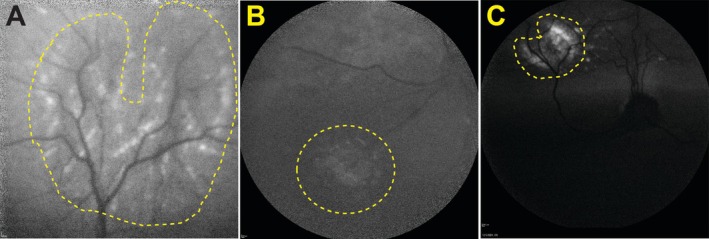
Autofluorescent confocal scanning laser ophthalmoscopy images (cSLO) of geographic RD‐like lesions in the CKCS and the Labrador Retriever. (A) The right eye (OD) of the CKCS shows a horseshoe‐shaped autofluorescent lesion around the dorsal venule in the tapetal region (yellow dashed outline). (B) The left eye (OS) of the CKCS displays minimal autofluorescence within the focal, circular hypopigmented region in the ventronasal non‐tapetal fundus (yellow dashed outline). The OS autofluorescence was less intense than in OD. (C) The OS of the Labrador Retriever shows bright horseshoe‐shaped autofluorescence within the geographic RD‐like lesion around the dorsonasal venule in the tapetal region (yellow dashed outline). The autofluorescence intensity is comparable to the OD lesion of the CKCS. CKCS, Cavalier King Charles Spaniel; cSLO, confocal scanning laser ophthalmoscopy; OD, right eye; OS, left eye; RD, retinal dysplasia.

The OD of the CKCS displayed a horseshoe‐shaped autofluorescent lesion along the dorsal venule (Figure 2A). The OS exhibited a circular lesion in the ventronasal non‐tapetal fundus, though autofluorescence intensity was less pronounced than the OD (Figure 2B). The OS of the Labrador Retriever revealed a horseshoe‐shaped autofluorescent lesion in the dorsonasal tapetal fundus, with a signal intensity similar to the OD lesion of the CKCS (Figure 2C).

#### 2.4.3 cSLO Infrared Reflectance Findings

**FIGURE 3:**
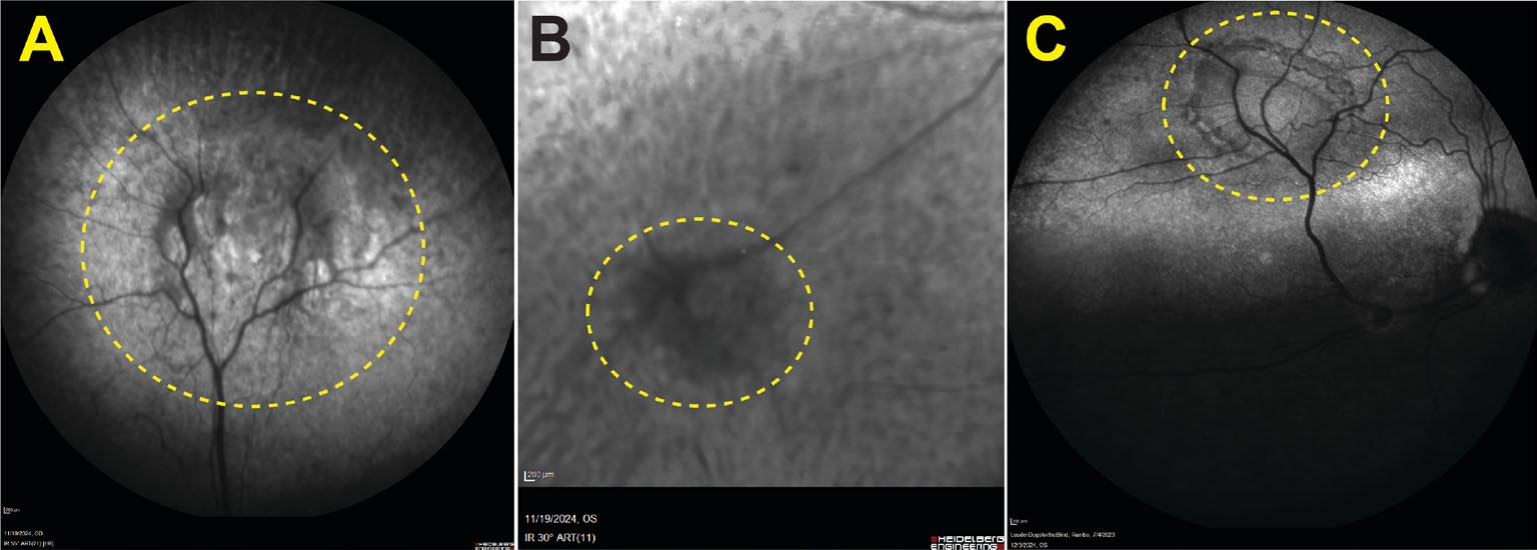
Infrared confocal scanning laser ophthalmoscopy (cSLO) images of presumed geographic RD in the CKCS and the Labrador Retriever (yellow dashed outline). (A) The OD of the CKCS exhibits minimal infrared (IR) reflectance over the horseshoe‐shaped lesion in the dorsal venule region, consistent with findings on other imaging modalities. (B) The OS of the CKCS also shows minimal IR reflectance over the circular lesion in the ventronasal non‐tapetal fundus, differing from OD. (C) The OS of the Labrador Retriever reveals minimal IR reflectance over the circular lesion in the dorsonasal tapetal region, resembling the OD lesion of the CKCS. CKCS, Cavalier King Charles Spaniel; cSLO, confocal scanning laser ophthalmoscopy; IR, infrared; OD, right eye; OS, left eye; RD, retinal dysplasia.

Infrared imaging of the OD of the CKCS showed minimal reflectance over the horseshoe‐shaped lesion (Figure 3A). The OS displayed patchy reflectance within the circular lesion in the non‐tapetal fundus (Figure 3B). Infrared imaging of the OS of the Labrador Retriever revealed minimal reflectance over the horseshoe‐shaped lesion in the tapetal fundus (Figure 3C).

#### 2.4.4 OCT Findings

**FIGURE 4:**
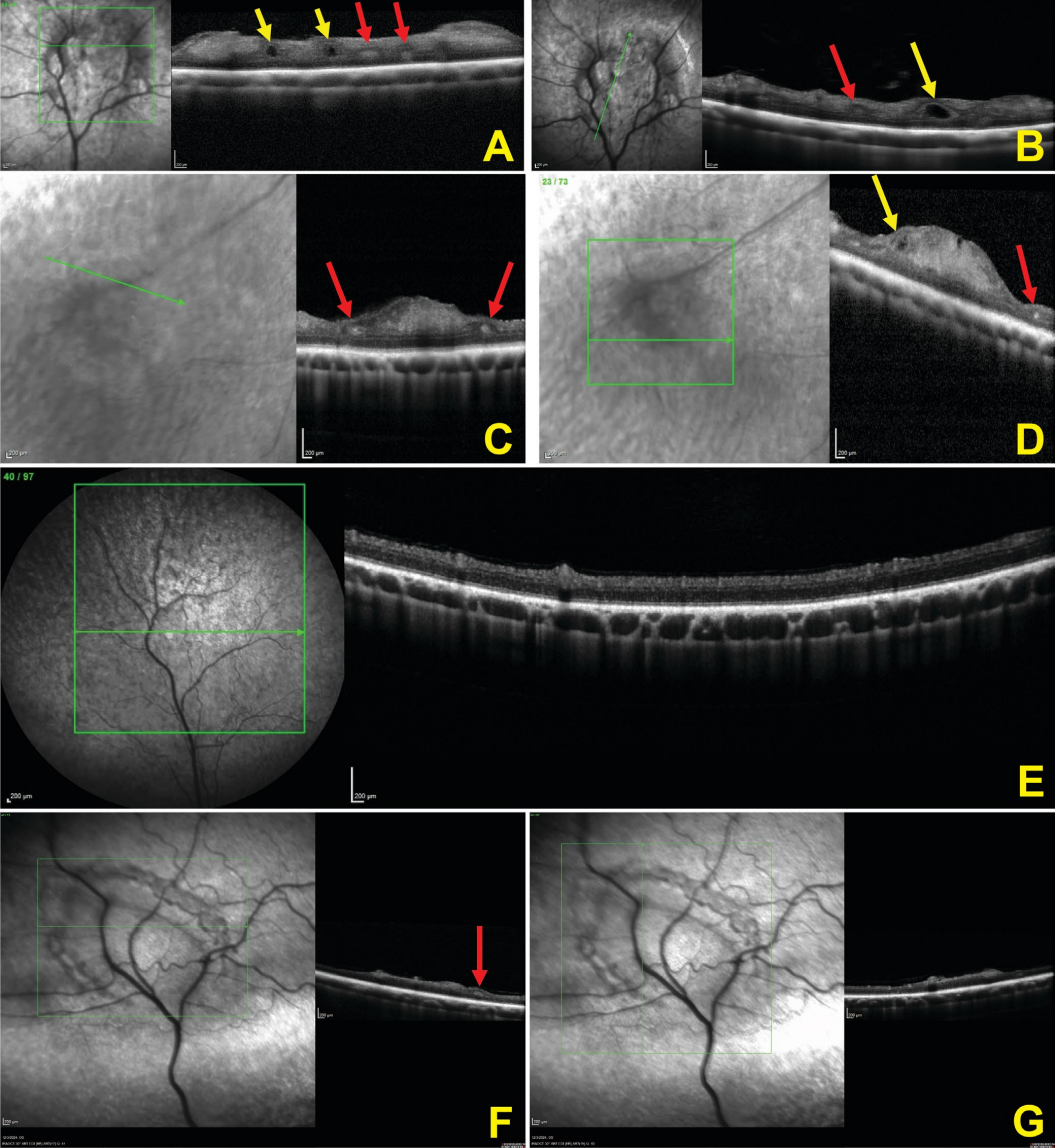
Comparative OCT and infrared en face images of retinal plaques in the CKCS and the Labrador Retriever. Images (A–D, F–G) were obtained using a 30° lens, while image (E) was captured with a 55° lens. In each panel, the green line with an arrow in the cSLO image on the left shows the plane displayed in the OCT on the right. Examples of suspected rosettes are indicated with arrows: Yellow for hyporeflective lumina and red for hyperreflective lumina. (A, B) The OD of the CKCS demonstrates a horseshoe‐shaped lesion with marked thickening of the retinal nerve fiber layer, consistent with a plaque. Within this plaque, multifocal hyperreflective lesions, some suspected to be outer retinal rosettes (red arrows), and multifocal hyporeflective lesions (yellow arrows) are evident. (C, D) The OS of the CKCS shows a circular lesion with findings similar to those of OD. Thickened retinal nerve fiber layers form a plaque‐like structure containing multifocal rosettes (red and yellow arrows). (E) Dorsal venule region of CKCS OS with normal retinal conformation, serving as a control for comparison with the retinal plaques. (F, G) The OS of the Labrador Retriever shows a circular retinal lesion with an associated plaque, though the thickness is less pronounced compared to the CKCS. Multifocal rosettes with hyperreflective content (red arrows) are present within the plaque, but not in the outer retina. CKCS, Cavalier King Charles Spaniel; cSLO, confocal scanning laser ophthalmoscopy; OCT, optical coherence tomography; OD, right eye; OS, left eye.

Imaging OD of the CKCS revealed marked thickening of the retinal nerve fiber layer, forming a plaque‐like lesion with multifocal either hypo‐ or hyperreflective lumina in the inner and outer retina, suspected to be rosettes (Figure 4A,B). Similar findings were observed in the non‐tapetal circular lesion OS, with thickening and rosettes (Figure 4C,D). Imaging OS of the Labrador Retriever exhibited moderate thickening of the retinal nerve fiber layer, forming a plaque‐like lesion with scattered rosettes limited to the inner retina (Figure 4F,G). There were fewer rosettes than in the CKCS lesions. Furthermore, no rosettes with hyporeflective lumina were observed, unlike in the CKCS.

Neither dog showed signs of focal separation of the neurosensory retina from the retinal pigment epithelium, which would suggest RD.

#### 2.4.5 OCTA Findings

**FIGURE 5:**
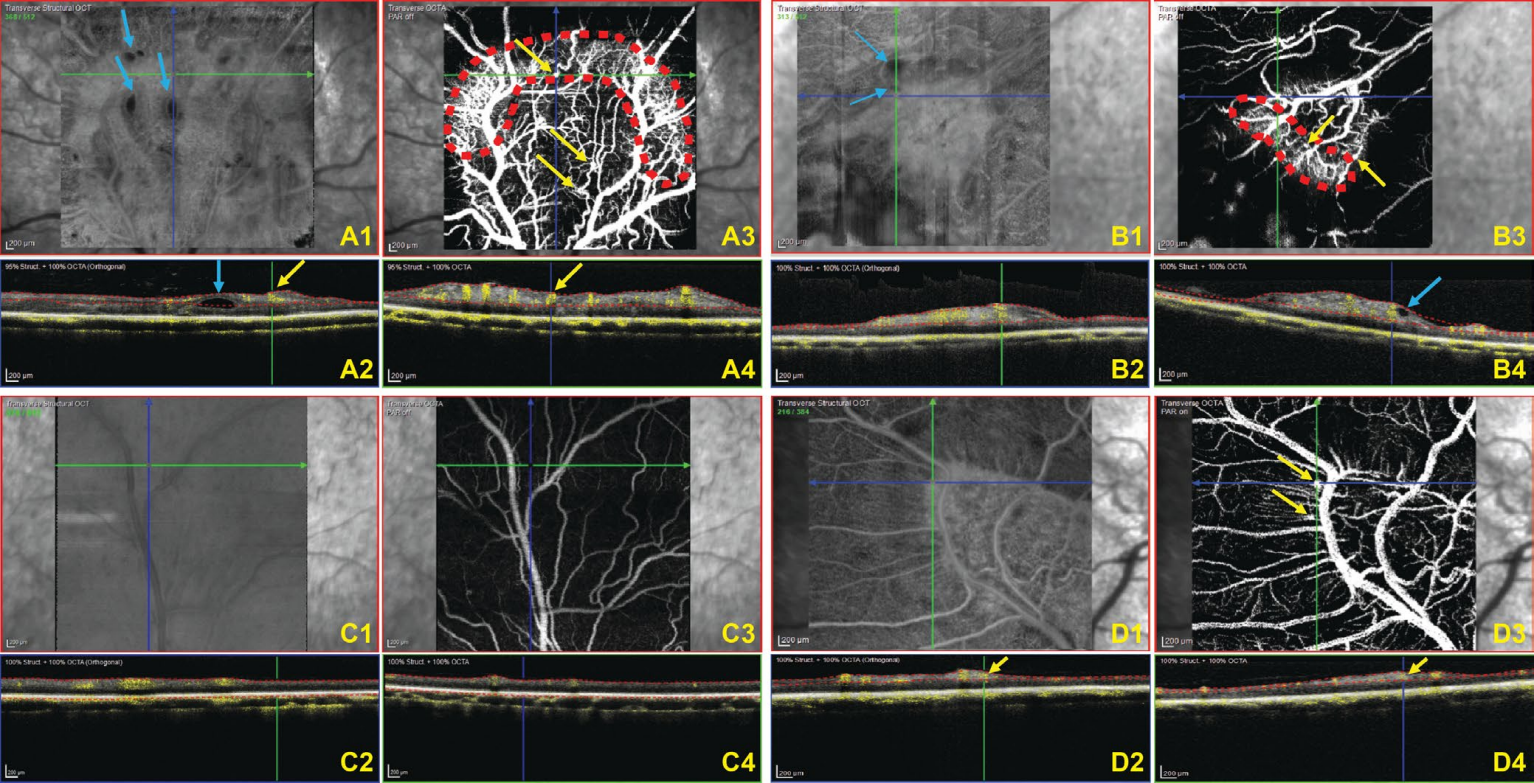
Comparative OCT angiography (OCTA, 30° lens) imaging in the CKCS and the Labrador Retriever, highlighting vascular and structural anomalies associated with the retinal plaques. (A‐D) Each subpanel shows the en face OCT image (top left: A1, B1, C1, D1) and OCTA angiogram of superficial/inner retinal vessels (top right: A3, B3, C3, D3) with the corresponding OCT/OCTA fusion images below with OCTA data as a yellow overlay (A2, A4, B2, B4, C2, C4, D2, D4: Yellow dots = blood flow). The green lines with arrows in the top left and right images (A1, A3, B1, B3, C1, C3, D1, D3) indicate the plane shown in the bottom left and right OCT/OCTA images (A2, A4, B2, B4, C2, C4, D2, D4). (A) The OD of the CKCS displays a dorsal venule lesion with suspected aneurysms (yellow arrows) visible in cross‐section and associated with concentrated blood flow. Some suspected aneurysms are visible within the peripheral highly vascularized region (dotted red outline). Multifocal hyporeflective lumina devoid of vasculature are noted (blue arrows), one of which is captured in cross‐section. (B) The OS of the CKCS displays a ventronasal lesion characterized by multifocal suspected aneurysms (yellow arrows) and hyporeflective lumina devoid of vasculature (blue arrows), one of which is seen in cross‐section. The peripheral highly vascularized region is outlined by the red dotted line. (C) The OS of the CKCS in the dorsal venule region exhibits normal vascular conformation and angiography findings. (D) The OS of the Labrador Retriever shows a dorsonasal lesion with suspected aneurysms (yellow arrows) visible in cross‐section, associated with affiliated blood flow. CKCS, Cavalier King Charles Spaniel; OCT, optical coherence tomography; OCTA, optical coherence tomography; OD, right eye; OS, left eye.

A plexus‐by‐plexus analysis of the superficial capillary plexus retinal vasculature was performed with OCTA for both cases. Imaging OD of the CKCS revealed multifocal rosettes with hyporeflective lumina (Figure 5A). The significance of these hyporeflective lesions is unknown. Multiple regions of the lesion contained these hyporeflective lumina. Within the geographic lesion, there were also suspected aneurysms with blood flow (Figure 5A). These suspected aneurysms exhibited noticeable tortuosity and were multifocal throughout the plaques. Similar, though less prominent, findings were noted in the retinal plaque OS (Figure 5B). In both eyes, the periphery of the retinal plaques contained a highly vascular region (dotted red outline in Figures 5A3 and 5B3).

The OCTA imaging of the Labrador Retriever revealed multifocal suspected aneurysms and vascular tortuosity within the lesion OS (Figure 5D). There were fewer suspected aneurysms in the lesion of the Labrador Retriever compared to the lesions of the CKCS. Unlike the CKCS, there were no hyporeflective lumina noted within the lesion of the Labrador Retriever. The OD and the non‐tapetal region OS were normal in retinal architecture and vascular supply (Figure 5C).

## 3 Discussion

This report represents the first detailed description of presumed geographic retinal dysplasia in a CKCS and provides additional documentation of inner retinal plaques in a Labrador Retriever using cSLO, OCT, and OCTA. The inner retinal plaques described in these two dogs, which cannot be differentiated from geographic RD without high‐resolution imaging, closely resemble those previously reported in Labrador Retrievers, Golden Retrievers, and German Shepherds [11, 13, 17, 18].

Both the CKCS and Labrador Retriever in this study exhibited significant retinal nerve fiber layer thickening, forming a plaque‐like horseshoe‐shaped retinal lesion along the dorsal venule, and an additional smaller, similar lesion along a ventral venule in the CKCS. All of the retinal plaques contained multifocal suspected rosettes. In addition, the CKCS also had suspected rosettes in the outer retina with the lesions. Some of these rosettes had hyporeflective lumina, while others had hyperreflective lumina. Others previously reported these different OCT appearances of rosettes [4]. Furthermore, similar retinal plaques with rosettes were previously reported in Labrador Retrievers, Golden Retrievers, and German Shepherds [11, 13, 17, 18].

Autofluorescence was noted in both dogs and appeared confined to the retinal plaque areas. The autofluorescent patterns in these dogs were similar to those previously reported by Ripolles‐Garcia et al. in 2022 [18] and by Iwabe et al. in 2020 [13]. Both reports included dogs with similar geographic RD‐like lesions that exhibited non‐uniform autofluorescence within the associated retinal plaques similar to those seen in our CKCS and Labrador Retriever. The focal areas of autofluorescence could represent macrophage migration and phagocytosis of cellular debris, which would have to be confirmed by histopathology [20].

Infrared reflectance was minimal within all our described inner retinal plaques. Similar to Iwabe et al. in 2022 [13], the plaque lesions appeared when imaged with infrared cSLO. In contrast to the previous report, we did not observe dark gray folds with infrared imaging in the CKCS and the Labrador Retriever. The minimal infrared reflectance observed in our case reports was likely due to retinal structural disruption [21].

Vascular anomalies were not readily visible via funduscopy, but were prominent on OCTA in both our cases. Suspected aneurysms and disruptions in the vascular plexus were observed, with the CKCS showing a higher density of vascular anomalies at the periphery of the lesions. The multifocal aneurysms in the CKCS and the Labrador Retriever were nearly identical to the ones described by Ripolles‐Garcia et al. (2022) in which multifocal aneurysms were noted within the retinal lesion of a German Shepherd with multifocal retinal dysplasia [18]. Histology with immunohistochemistry may provide more details as to the pathogenesis and process of these retinal lesions. Serial imaging would also help monitor the potential progression of the lesions over time, in addition to visual status.

A major limitation of any case report is the small number of cases, which limits the ability to draw conclusions for the larger canine population. For example, variations in plaque features among dogs likely reflect differences in lesion severity or individual variability rather than breed specificity. Nevertheless, our case report suggests that the presumed geographic RD observed in some CKCS may contain inner retinal plaques resulting from thickening of the retinal nerve fiber layer. Furthermore, we describe an additional Labrador Retriever with vascular retinal plaques, supporting previous publications on this condition in the breed [13, 17, 18]. Another limitation of our report is the missing ophthalmic examinations before the age of 4 months. This limited our ability to determine whether the retinal lesions developed during retinal development or whether they emerged later and could be acquired.

In summary, we provide further evidence that in at least some dogs, retinal lesions appearing like geographic RD on ophthalmoscopy may contain inner retinal plaques; an observation that can only be made with high‐resolution imaging.

## Author Contributions

**Shaile Gehrke:** conceptualization, data curation, formal analysis, investigation, methodology, visualization, writing – original draft, writing – review and editing. **Christine D. Harman:** formal analysis, investigation, methodology, visualization, writing – review and editing. **Amanda L. Jacobson:** investigation, methodology, writing – review and editing. **Gail McRae:** conceptualization, investigation, methodology, writing – review and editing. **András M. Komáromy:** conceptualization, data curation, formal analysis, funding acquisition, investigation, methodology, resources, supervision, validation, visualization, writing – original draft, writing – review and editing.

## Funding

This work was supported by the National Eye Institute (Grant R01‐EY032478) and BrightFocus Foundation (Grant G2022007S).

## Disclosure

Artificial Intelligence Statement: The authors did not use AI to generate any part of the manuscript. AI tools were utilized to enhance spelling, grammar, and overall editing.

## Ethics Statement

This study adhered to the Guidelines for Ethical Research in Veterinary Ophthalmology (GERVO) and the Association for Research in Vision and Ophthalmology (ARVO) Statement for the Use of Animals in Ophthalmic and Vision Research. Dog owners provided written consent for all diagnostics and publication of results and images.

## Conflicts of Interest

The authors declare no conflicts of interest.

## Data Availability

The data that support the findings of this study are available from the corresponding author upon reasonable request.
